# Global Collembola on Deception Island

**DOI:** 10.1673/031.012.11101

**Published:** 2012-10-05

**Authors:** Penelope Greenslade, Mikhail Potapov, David Russell, Peter Convey

**Affiliations:** ^1^ Environmental Management, School of Science and Engineering, University of Ballarat, PO Box 663, Mt Helen, Victoria, Australia 3350; ^2^ Department of Zoology and Ecology, Moscow State Pedagogical University, Kibalchich str., 6, korp. 5, Moscow 129164, Russia; ^3^ Dept. Soil Zoology; Section Mesofauna, Senckenberg Museum of Natural History Görlitz, Postfach 30015, 02806 Görlitz, Germany; ^4^ British Antarctic Survey, High Cross, Madingley Road, Cambridge CB3 0ET, UK

**Keywords:** human-mediation, invasive species, non-indigenous species, springtails, tourist impacts

## Abstract

Three new non-indigenous springtail species are recorded in recent collections made on Deception Island, South Shetland Islands, maritime Antarctic: *Deuteraphorura* (*Deuteraphorura*) *cebennaria* (Gisin) (Collembola: Onychiuridae), *Mesaphorura macrochaeta* Rusek (Tullbergiidae), and *Proisotoma minuta* Axelson (Isotomidae). One of these, *D.* (*D*.) *cebennaria*, is described. Additionally, two new indigenous species, *Mesaphorura macrochaeta* Rusek and *Proisotoma minuta* Axelson, are also recorded. The total number of Collembola species now known from the island is 14, comprised of eight native species and six non-indigenous species. This number of non-indigenous species recorded at Deception Island compares with only a single non-indigenous springtail recorded at any other maritime or continental Antarctic location. The reason underlying this high level of occurrence of non-indigenous species on Deception Island is likely to be a combination of the island's high level of human visitation and the presence of relatively benign terrestrial habitats associated with areas of geothermal activity. Two of the new records represent species recently assessed as being of the highest risk to become invaders in the less extreme environments of the subantarctic, thereby emphasising the importance and urgency of adopting and applying effective biosecurity measures to protect the unique and vulnerable ecosystems of this region. Also documented are the impacts on the soil fauna of the island from human trampling, which drastically reduced densities of both native and non-indigenous species to 1% of the abundance typical of non-trampled sites.

## Introduction

**Figure 1.**
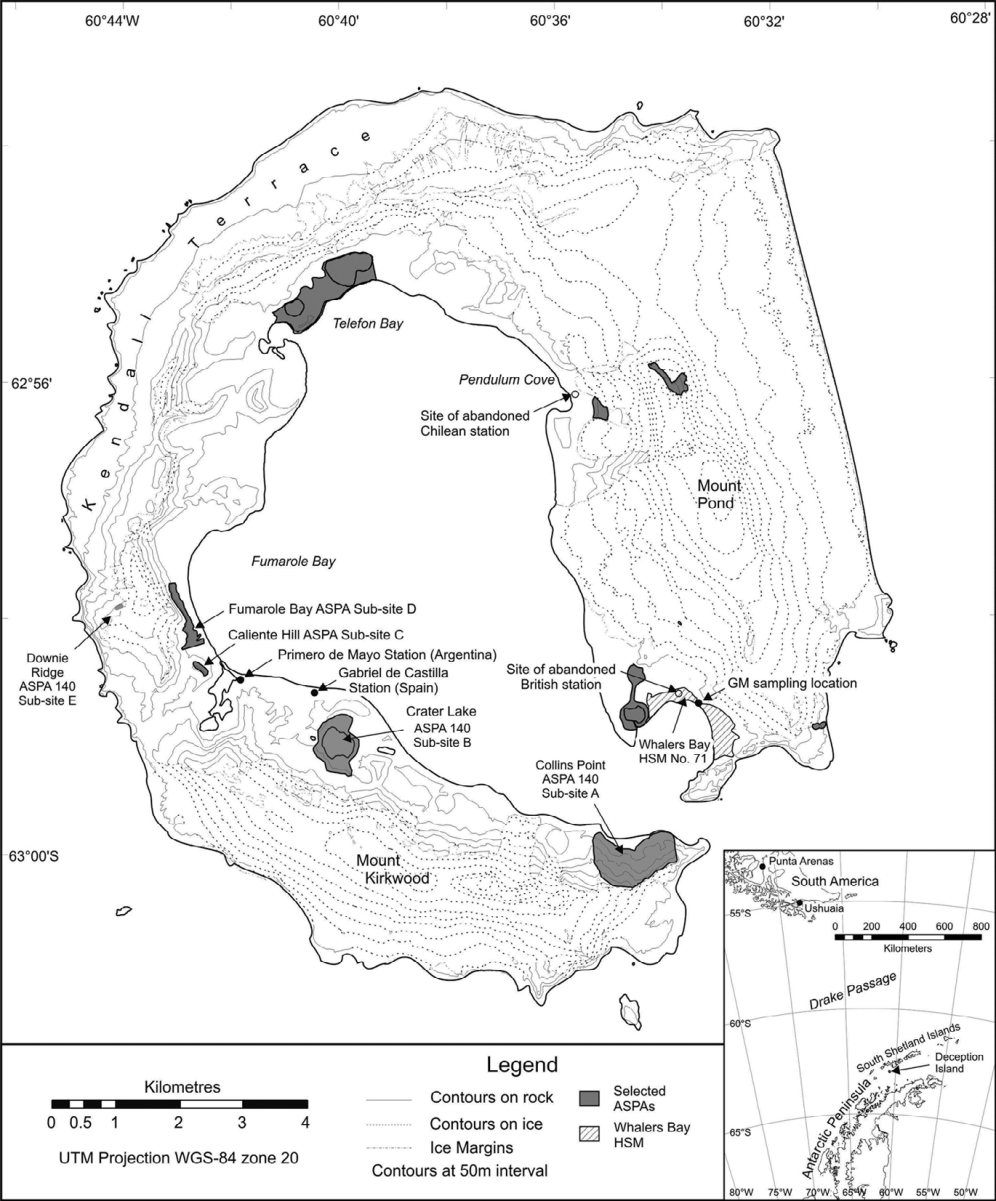
Map of Deception Island, including (inset) the island's location in the South Shetland Islands, showing the different areas where collections were made in the current study. High quality figures are available online.

Deception Island (62° 57′S 60° 38′ W) lies in the South Shetland Islands archipelago, northwest of the tip of the Antarctic Peninsula, in the southern part of the Scotia arc (Figure 1). It consists of an active caldera whose last major eruptions, which caused considerable damage, occurred between 1967 and 1970 (Baker et al. 1975). The caldera is open to the sea, because of the horseshoe shape of the island, and so provides sheltered anchorage for vessels. Consequently, the island has been among the most visited regional localities by explorers, sealers, whalers, tourists, and scientists over the last 200 years (Convey and Lebouvier 2009). Norwegian whalers occupied the island during the early part of the Twentieth Century, constructing a shore-based whaling station. At that time, 13 ships could be anchored at the station at once (Hart 2006).

The island was the site of three scientific stations operated by the United Kingdom, Chile, and Argentina prior to the most recent volcanic eruptions. During the eruptions, the two former stations were extensively damaged or destroyed, and subsequently abandoned. At present, Argentina and Spain operate summer only research stations there. The island has had one of the highest visitation rates in the Antarctic by tourist vessels, although the tourism is focused on a limited number of sites. For instance, over a period 24 days in February 2002, an average of nearly three vessels were at the island on any one day (Roura et al. 2008). Whalers Bay, the site of the former whaling station and abandoned British research station and airstrip, is one of the most visited sites in the entire maritime Antarctic region, with more than 16,000 tourists reported to have landed between 2009 and 2010 (Lynch et al. 2010; IAATO 2011).

Deception Island is designated an ‘Antarctic Specially Managed Area’ under the Antarctic Treaty System, and has an international management committee. Within the Antarctic Specially Managed Area, parts of the island are further protected as Antarctic Specially Protected Areas (ASPA). A management plan, which included a code of conduct for all visitors to the island, was prepared in 2005 (Anonymous 2005a,b). Visitor site guidelines exist for Telefon Bay, Baily Head, and Whalers Bay, but other sites, including Pendulum Cove, are also regularly visited. A brief history of Deception Island and a review of its fauna and flora was given by Downie et al. (2000).

The active volcanic nature of the island, in particular the existence of various types of geothermally-influenced habitats (warmed ground, fumaroles) (Smellie et al. 2002), means that its terrestrial biology is exceptional in many respects for the Antarctic (Smith 2005a,b; Convey and Smith 2006). However, only a very small proportion of the terrestrial habitats are subject to geothermal influence, and much of the island's area is covered by unstable and largely barren volcanic ash and scoria (Smellie et al. 2002). Wherever terrestrial biota become established on unheated areas, communities typical of the maritime Antarctic develop on ground and rock surfaces that are sufficiently stable. In contrast, geothermally warmed habitats, which are only found in the maritime Antarctic on Deception Island and in the South Sandwich Islands, host a range of native plant and invertebrate species that are otherwise unknown in the Antarctic (Aptroot and van der Knaap 1993; Convey et al. 2000a,b; Convey and Smith 2006).

Deception Island's terrestrial fauna and flora, including the Collembola, are thought to be relatively well known (Smith 1984, 1988, 2005a,b; Aptroot and van der Knaap 1993; Downie et al. 2000; Izaguirre et al. 2006); however, much of the island still remains unsurveyed. Nine Collembola species were known until now to inhabit the island, including three possibly invasive nonindigenous species (NIS) (Table 1; definitions of the terms non-indigenous, exotic, introduced, and invasive are given in Greenslade and Convey (2011)) (Greenslade & Wise 1984; Downie et al. 2000). Hack (1949) was the first to record a NIS, *Hypogastrura viatica* (Tullberg), from under a whale carcass lying on warmed soil. Wise (1971) later recorded the same species from Tower Island, Palmer Archipelago (c. 63° 34′ S), and Greenslade (1995) extended its distribution further south by recording it on Léonie Island, near Adelaide Island (c. 67° 36′ S). Two new records of this species are from Half Moon Island in the South Shetland Islands group (62° 35′ S) and Neko Harbour in Graham Land (64° 50′ S) (M. P., unpublished data). Both sites are regular tourist visitation sites. The two other NIS, *Folsomia Candida* Willem and *Protaphorura fimata* (Gisin), reported previously from Deception Island, were also found under whale bones on a geothermally active beach (Greenslade and Wise 1984; Greenslade 2010). The existing published total of three NIS was the highest recorded for any location in the maritime or continental Antarctic. It is only paralleled or exceeded in the wider Antarctic region by some subantarctic islands (South Georgia (4 species), Macquarie Island (11), Marion Island (5), Îies Kerguelen (6), Îies Crozet (3)) (Greenslade and Convey 2011). Important factors that have been proposed as underlying the colonization of Deception Island by non-indigenous Collembola include the availability of habitats subject to permanently warm (and moist) conditions and the high rate of human visitation (Downie et al. 2000).

Two new collections of Collembola were made from the island during the austral summer in January 2010. One collection, by British Antarctic Survey (BAS) personnel operating from the Spanish Gabriel de Castilla research station, included samples obtained from both pristine sites (including specific geothermally influenced and non-heated areas) and from areas of current and historical human impact. The second collection was made by a German expedition collecting only from an area of high tourist visitation both currently and in the past. In this study, we have documented new records of NIS of Collembola for Deception Island, from both recent collections as well as two new records of indigenous species. We also provide brief observations on the effect trampling has on native as well as NIS soil-inhabiting Collembola.

## Materials and Methods

The first series of samples were obtained between 19 and 24 January 2010 by P. Convey and KA. Hughes (BAS) and consisted of 63 samples from eight distinct areas on Deception Island (Figure 1; see Anonymous (2005a) for detailed descriptions of the various sub-sites of the Deception Island ASPA). Samples obtained from Caliente Hill (ASPA sub-site C) and Downie Ridge (ASPA sub-site E, previously known as West Stronethrow Ridge) included mosses growing on actively heated ground in areas that are currently of very restricted human access. Those from Crater Lake (ASPA sub-site B), Fumarole Bay (ASPA sub-site D), and Collins Point (ASPA sub-site A) included mosses and lichens currently growing on unheated ground. Finally, samples from Whalers Bay (throughout the area of HSC No. 71, to its boundary with ASPA sub-site K), the vicinity of the Argentinean summer-only Decepción station, and Pendulum Cove (below ASPA subsite G) included mosses, lichens, and algae collected from both the vicinity of active or abandoned buildings and sites of tourist visitation. None of the collection localities at the latter two sites showed signs of current geothermal influence, although beaches at both Whalers Bay and Pendulum Cove are warmed and, as a result, are two of the most popular tourist landing locations.

**Table 1.**
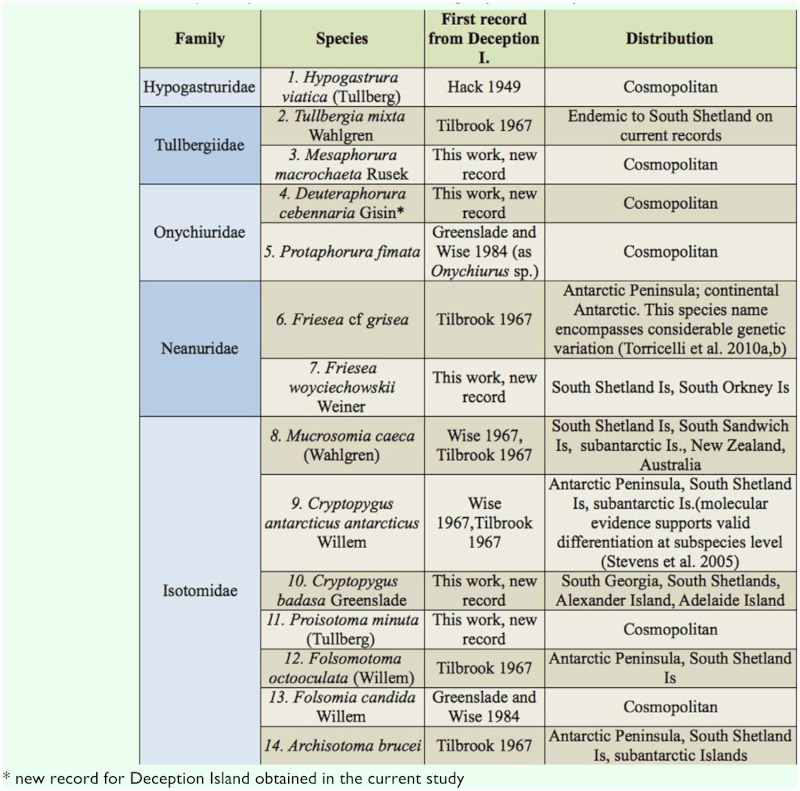
List of Collembola species currently recorded from Deception Island, with source references. Total listed = 13 (6 exotics, 7 confirmed native). One possible additional native, single specimen only found.

Arthropods were obtained by Tullgren extraction of the samples of moss and lichen vegetation, carried out within 2–3 days after collection at the Spanish Gabriel de Castilla station. Samples from all 63 extractions were examined superficially for the presence of NIS springtails. Species identifications of springtails in 20 collections were made by P. Greenslade at the BAS laboratories in Cambridge, United Kingdom, in October 2010.

The second series of samples were taken by A. Kertelhein (on behalf of the Senckenberg Museum of Natural History Görlitz, Germany) (GM) on 19 January 2010. He took four soil cores of 5.5 cm diameter and 5 cm depth from each of four sites at Whalers Bay (Figure 1). Two sites were in an area subject to trampling by visitors, and two, slightly upslope from the first two, were not trampled (Figure 3). The sites were all within 100 m of each other. Collembola were extracted in Germany from the cores by using a Macfadyen-type high-gradient extractor on return of the samples to Germany. These soils were dark, and absorbed and retained solar heat energy. Temperatures measured with a hand held digital “Precision” thermometer at the time of collection were low, with no suggestion of active geothermal influence (mean 10.6° C, range 9.2 to 13.0). Soil moisture content had a mean of 14% dry mass (range 11.9 to 15.8%), and the pH was acidic (mean 4.58, range 3.8 to 6.9).

Sample collections, curation, and extractions were carried out in such a way as to avoid the risk of contamination. The BAS samples were extracted using cleaned equipment as soon as practical after collection on Deception Island, and the extracted alcohol-preserved samples were not re-opened until examination. The GM soil core samples were packed within several layers of plastic, and transported under refrigerated conditions to Germany, where they were extracted nine days after collection under quarantine conditions.

## Results

Eleven species of Collembola were collected in the BAS samples, including one previously unrecorded NIS (Table 1). Although the extraction technique used did not permit rigorous quantitative comparisons, most native species were abundant and frequent in collections. They included *Folsomotoma octooculata* (Willem), *Tullbergia mixta* Wahlgren, *Cryptopygus antarcticus* *antarcticus* Willem, *Cryptopygus badasa* Greenslade, and *Friesea* aff. *grisea* Schäffer. The NIS *H. viatica* was also abundant in some collections. *Friesea woyciechowskii* Weiner, *P. minuta* (Tullberg), and *Mucrosomia caeca* (Wahlgren) were rare or more localised. Neither of the other previously recorded NIS, *F. Candida* and *P. fimata*, were present in the collections. The only other maritime Antarctic records of *M. caeca* are from heated areas of the South Sandwich Islands (Convey et al. 2000a), although the species is also present further north in the Scotia arc on subantarctic South Georgia, the type locality (Convey et al. 1999), in other subantarctic islands, and in Australia and New Zealand. The GM samples included six species, of which three were considered native, three were NIS, and two of the NIS were new for Deception Island. The last two NIS species are already known to have invaded other subantarctic islands (Table 1). Differences in Collembola abundance between trampled and non-trampled areas in Whalers Bay (GM collections) were very large (Mann-Whitney U = 10.667, *p* = 0.001; Table 2). The mean collembolan densities in the eight cores obtained were equal to 360,000 m-2 in the non-trampled areas and 10,400 m-2 in the trampled areas. Extractions from the non-trampled area included only one native species, *C. antarcticus*, and one NIS, *H. viatica*, but six species, including three NIS, were found in the trampled area. The densities of both the numerically dominant NIS and the native *C. antarcticus* were affected equally, being reduced to less than 1% of the levels seen in the non-trampled areas (Table 2).

**Table 2.**
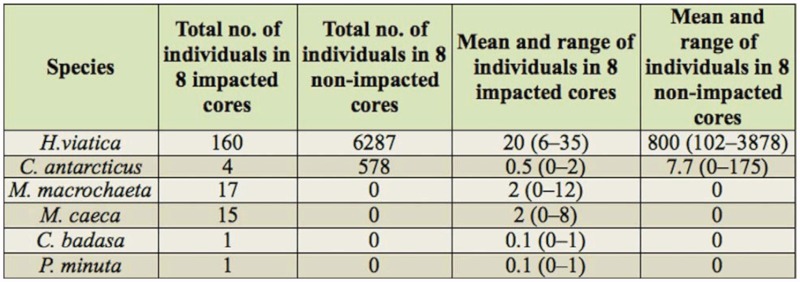
Abundance of Collembola species in trampled (n = 8) and non-trampled (n = 8) soil cores obtained at Whalers Bay in January 2010.

### New records of non-indigenous species

Details are given below for the NIS Collembola and the two indigenous species that are new records for Deception Island. Taxonomic and ecological notes on the other species recorded can be found in the citations given in Table 1. A more detailed description is given for one of the new NIS records, that of *Deuteraphorura* (*Deuteraphorura*) *cebennaria* (Gisin, 1956) (Onychiuridae), to enable easier identification of any future collections.

### Onychiuridae

*Deuteraphorura* (*Deuteraphorura*) *cebennaria* (Gisin, 1956) *sensu* Fjellberg 1998 and Pomorski 1998.

**Figure 2.**
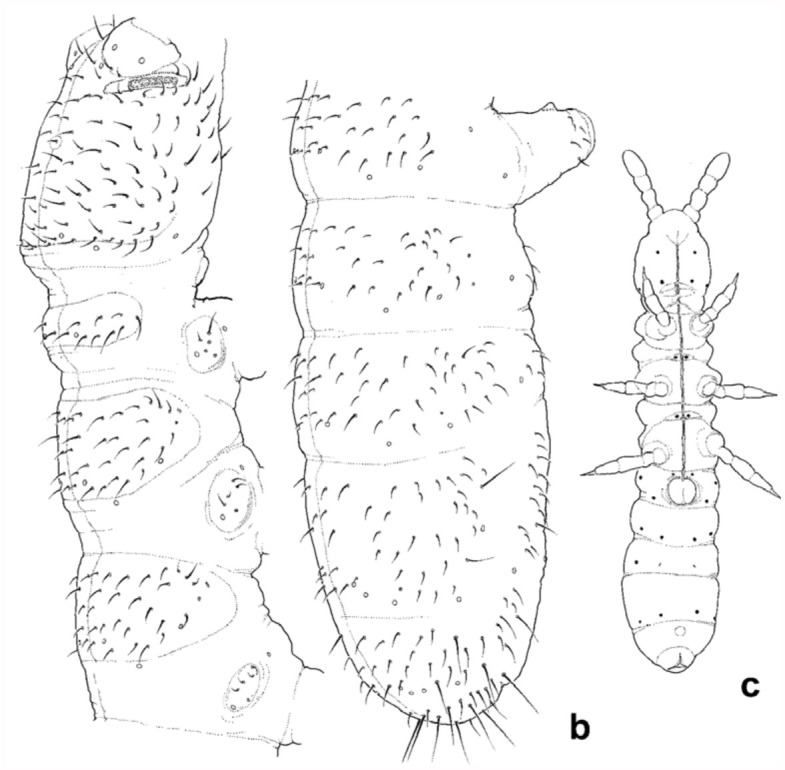
(a) *Deuteraphorura cebennaria* (Gisin, 1956) *sensu* Fjellberg 1998 and Pomorski 1998, dorsolateral view of head and thorax showing chaetotaxy and arrangement of pseudocelli; (b) *Deuteraphorura cebennaria* (Gisin, 1956) *sensu* Fjellberg 1998 and Pomorski 1998, showing dorsolateral view of abdomen, c) ventral view showing arrangement of pseudocelli and parapseudocelli. High quality figures are available online.

**New record** (Figure 2a,b,c).

Material examined: 1 subadult male, 1 adult female. Nine other specimens in alcohol. Locality data: Deception Island, Pendulum Cove, near and on ruins of Chilean Station, in moss *Polytrichum, Pohlia*, 20 January 2010, P. Convey, sample 25.

### Description

Length 1.5– 1.6 mm. PAO, as common for this group, with 13–17 complex vesicles. Antennal organ with 5 simple papillae, 2 rods, and 2 smooth curved sensory clubs. Dorsal pseudocelli: 32/133/33354 (male with only 4 pseudocelli on one side of Abd. V), positioned as common for the genus. Ventral pseudocelli: 3/011/3212. Subcoxae with 2 pseudocelli on each leg (2,2,2). At least a pair of parapseudocelli present on Abd III, in medial position (Figure 2a). Other parapseudocelli were not observed. Setae on body short, macrosetae well developed only distally on abdomen. Apical setae of abdomen slightly bifurcated at tip. Chaetotaxy of subadult male illustrated in Figures 2a,b. Ventral tube with about 6 + 6 distal setae. Abd. IV with unpaired seta m0, setae ml present. Medial macrosetae M less than twice as long as adjacent sensilla (s) on Abd. V. Abd. VI with asymmetrical chaetotaxy (in specimens examined) in medial part. Four microsetae behind furcal field in irregular position. Inner tooth on claws absent, lateral teeth vary (conspicuous on some legs). Unguiculus gradually tapering. Fully reproductive males (with *ductus ejaculatorius*) not found. Large subadult male under study lacking modified setae on ventral part Abd. II or Abd. III.

### Taxonomic and habitat notes

The species belongs to the tribe Onychiurini (Pomorski 1998) because posterior pseudocelli on the head are present, the postantennal organ has numerous complex vesicles, and the furca is reduced to a finely granulated area with 2 + 2 or 1 + 1 setulae in a single row. The specimens possess the characters of the genus *Deuteraphorura* Absolon *sensu* Pomorski, 1998 with seta d0 present on head, 9 setae in distal whorl of tibiotarsi, PAO with compound tubercles, anal spines absent, and antennal organ with 5 papillae. The genus *Deuteraphorura* is the second largest genus (after *Protaphorura*) of Onychiurinae, with 72 species recognised (Bellinger et al. 2011). The members of the genus are mainly found in European caves, particularly in France, Switzerland, and Italy, with some species also living above ground in deep forest litter and composts, the same habitats occupied by *D. cebennaria* according to Fjellberg (1998). Several other *Deuteraphorura* (*D*.) species have been introduced to parts of Europe and elsewhere outside their natural range, as have other species of Onychiurinae, including *Deuteraphorura variabilis* (Stach), *Orthonychiurus folsomi* (Schäffer), and *O. stachianus* (Bagnall). They all inhabit disturbed biotopes in Europe.

The specimens examined have an identical morphology to *D*. (*D*.) *cebennaria* redescribed by Pomorski (1998) and Fjellberg (1998), except that p0 on abdomen IV is the same length as p1, while it is longer in the descriptions of this species by these two authors. *Deuteraphorura cebennaria* was originally described as *Onychiurus cebennaria* by Gisin (1956) from a cave in France (Ardèche), and was characterized by the ventral organ being missing in males (present in Polish and Scandinavian specimens), and, according to Dallai (1969), setae ml on Abd. IV absent (present in Deception Island specimens). The Deception Island specimens could possibly be identified as *Deuteraphorura difficilis* (Dallai, 1969) described as living in leaf litter in Italy. We consider that *D. difficilis* and *D.* (*D*.) *cebennaria* may be synonymous. *Deuteraphorura bangazeya* Babenko from Siberia is also similar to the Deception Island specimens. It is the most northerly species of the genus, with dorsal pseudocelli as 32/133/33353 and ventral as 3/011/3212, indicating that the number of pseudocelli on Abd. V provides a distinct difference (3 *cf*. 4), although some variability in this character (3–4 pso on Abd. V) was mentioned by Dallai (1969) for *D. difficilis*. There are several names in older literature for species lacking anal spines with which *D. cebennaria* may be synonymous. As available descriptions of these species are inadequate, it is impossible to ascertain correctly their characters. One of these is *Lipura inermis* Tullberg from Scandinavia, which probably also belongs to the genus *Deuteraphorura* (Janssens 2011).

Eleven specimens of this species were present in one of the 63 BAS extractions taken from a clump of *Polytrichum alpinum* at Pendulum Cove. This is an area that was heavily impacted by the 1968 eruptions, when the Chilean station at this site was evacuated, destroyed, and not reoccupied. Currently, Pendulum Cove is a popular tourist visitation site, as the beach is strongly heated and steaming over much of its length, and the shallows are very warm and often used as a bathing area. There is also a geophysical observing station installed near to the station ruins. There is no visible evidence or suggestion of active geothermal heating between the immediate beach area and the site of the ruined station buildings. A distinct patch of turf of *P. alpinum* was present on the flat area between the station ruins and the beach, but the area was otherwise largely unvegetated. In the same sample were specimens of the native *M. caeca, C antarcticus antarcticus*, and *F. octooculata*.

### Tullbergiidae

***Mesaphorura macrochaeta* Rusek, 1976**
**New record**

### Taxonomic and habitat notes

Seventeen specimens were obtained in extractions from trampled sites in the GM collections at Whalers Bay (Table 2). The species was not present in the 15 BAS collections obtained from the vicinity of Whaler's Bay, indicating that it is likely to currently have a very restricted distribution in this area. The morphology agrees with the most recent description of this species (Fjellberg 1998; Dunger and Schlitt 2011). *M. macrochaeta* inhabits a large range of macrohabitats, from undisturbed forests to arable fields, and is widespread in temperate climatic zones, reaching high latitudes of the Southern Hemisphere and Macquarie Island in the subantarctic (Greenslade 1992). It has demonstrated an ability to spread to northern high latitudes also, being recorded from Spitsbergen (Fjellberg 1994), and even in marine littoral habitats on Wrangel Island and in the Canadian High Arctic (Babenk and Fjellberg 2006). As a minute, exclusively soilinhabiting species, *M. macrochaeta* was probably originally introduced to the Southern Hemisphere and northern sites by human intervention in importations of soil and moss peat (Greenslade 2006).

### Isotomidae

***Proisotoma minuta* Tullberg, 1871**
**New record**

### Taxonomic and habitat notes

Several specimens were found at Caliente Hill from a warmed area (BAS sample 17), and a single specimen was obtained from a trampled site at Whalers Bay (GM collection). This species is cosmopolitan in distribution, but is normally found in habitats with higher organic matter content than the soil-inhabiting *M. macrochaeta*. It is more frequent in southern areas, including the tropics (Potapov 2001). It also has become naturalized on Macquarie Island, where it is thought to have been introduced along with *M. macrochaeta* in moss peat imported for growing vegetables in the now-removed greenhouses (Greenslade 2006). It is not found in the Arctic but does occur in northern Norway (Fjellberg 2007).

### New records of indigenous species

### Neanuridae

*Friesea woyciechowskii* Weiner, 1980

### Taxonomic notes

The species was originally described from King George Island, South Shetland Islands, from only two specimens (Weiner 1980), but is also known to be from the South Orkney Islands (Booth and Usher 1984). A single specimen was found in BAS sample 21 (possibly *Cephaloziella* sp.) from the eastern end of Crater Lake. Other species in the same sample were *T. mixta* and *F. octooculata*.

### Isotomidae

***Cryptopygus badasa* Greenslade, 1995**
*Habitat notes*.
  This species was described from Livingston Island, South Shetland Islands, and found to be common and abundant in Deception Island samples (10 out of 20 BAS samples examined in detail). It is also known from Adelaide Island, Alexander Island, and South Georgia (Greenslade 1995, 2010).

## Discussion

Greenslade and Wise (1984) recorded six species of Collembola from Deception Island, including two NIS, while Downie et al. (2000) recorded four species, leading to a total of nine species being known from the island, including Wise's (1967) and Tilbrook's (1967) records. The current study adds a further five, bringing the total to 14, and including six NIS. The number of NIS represents over one-third of the total terrestrial collembolan fauna known from Deception Island, although, apart from *H. viatica*, all the NIS appear to be restricted in distribution. *H. viatica* was found in four samples in the BAS collections, abundantly in two from Whalers Bay, as in the GM records, but also in small numbers from Collins Point and Caliente Hill. The species is therefore now distributed around the caldera, with two of these locations currently having tightly restricted human access. The high collembolan species richness is exceptional for an island so far south and includes all species known from the entire South Shetland archipelago (Greenslade 1995, 2010). Furthermore, apart from *H. viatica*, there are no NIS Collembola recorded from any other island in the South Shetlands group or from elsewhere in the maritime Antarctic.

We attribute the high number of NIS not only to the warmer and moister conditions available at some locations as a result of geothermal activity, but also to the continuous human presence on the island over a long period, and the current high level of tourism and national operator visitation to the island. No specific introduction event to Deception Island has been documented, as is to be expected in a region where the majority of the native flora and fauna are non-charismatic species, often with cryptic habitats, and likely to be overlooked in the absence of a specialist study (Hughes and Convey 2012). Two of the new NIS records were only collected from areas (Whalers Bay, Pendulum Cove) that have suffered and continue to experience considerable cumulative human impact. The third new NIS, *P. minuta*, was collected from Whalers Bay as well, but was also present in reasonable numbers in extractions of geothermally-influenced vegetation collected within the ASPA subsite at Caliente Hill, as was *H. viatica*. This site now has strict human access restrictions, although it is also a location close to the Argentinian research station and is a site of regular geological field studies. These access restrictions were fist imposed in 1985, when it was declared a Site of Special Scientific Interest (the predecessor to the current ASPA designation system), and only apply to the small area of the heated summit ridge of the hill.

Most collections from Deception Island have been serendipitous, and the exact sites of earlier records have not been re-sampled, so it is not possible to describe most recorded NIS as invasive. The exception to this generalisation is *H. viatica* because of its high abundance in the 2010 samples from Whalers Bay, contrasting with the apparent low density and few individuals reported there in the past (Wise 1967, 1970), and together with the new records from Collins Point and Caliente Hill. In a risk assessment of Collembola species likely to invade subantarctic South Georgia, *P. minuta* and *M. macrochaeta* were identified as being of the highest risk status (Greenslade and Convey 2011). It is significant that these two species have now been found considerably further south on Deception Island. For most introductions of nonindigenous species such as are described here, the practicalities of population monitoring are likely to be insurmountable given the resources available to operators in the region. This also means that eradication attempts applied to such species and monitoring the efficacy of such attempts are likely to be ineffective (Hughes and Convey 2012).

No NIS are currently known among the Acari that have been recorded from the island (or any other location in the maritime Antarctic); this absence is likely a reflection of the lack of survey effort, and several possible taxa are present in the GM samples obtained here (D. R., unpublished data). There was a recent instance of a new plant record near to the visitor-accessible area at Whalers Bay (Smith and Richardson 2010), which was removed as an application of the ‘precautionary principle,’ although definitive evidence of it being a human-assisted or natural colonization event could not be obtained (Hughes and Convey 2012). Many of these introductions would have occurred before any quarantine advice was developed for the island, such as the management plan prepared in 2005.

Present records of NIS from Deception Island are consistent with one of the conclusions of Gabriel et al. (2001) that the likelihood of a community being invaded depends, at least partly, on temperature. In contrast, Terauds et al. (2011) found no difference in the distributions of NIS and native collembolan species with environmental factors on subantarctic Macquarie Island, although their analysis did not take into account the different biologies and biogeographic affinities of native species, and they failed in several instances to distinguish native from exotic Collembola. Greenslade (2006) reported that, although species richness is lower at the higher altitudes on Macquarie Island compared to coastal sites, the suite of species at the former included no NIS, and the native species occurring there had affinities with other subantarctic islands to the west. In contrast, coastal sites harboured every NIS recorded from the island, and the native species found there predominantly had affinities with New Zealand's southern islands to the east.

The biology of each species is also likely to have played a role in their ability to colonise Deception Island. In this respect, it is significant that three of the NIS are parthenogenetic (*M. macrochaeta, P. fimata, F. Candida*). Chahartaghi et al. (unpublished) have demonstrated that parthenogenetic species of Collembola colonise vacant areas more quickly than those capable of sexual reproduction. *H viatica* is not parthenogenetic, but is known to have established and become invasive on several subantarctic islands, and has been recorded from elsewhere in the maritime Antarctic (Greenslade 1995; Frenot et al. 2005; Greenslade and Convey 2011). It appears able to outcompete the native *C. antarcticus antarcticus* on coastal sites on South Georgia (Convey et al. 1999).

The practically complete elimination of Collembola from areas subject to human trampling within the popular visitor site at Whalers Bay is notable. Sands et al. (1979), studying compaction of soils in pine forests in South Australia, noted that the first passage contributed the major increase in soil density. In the continental Antarctic (Victoria Land Dry Valleys), Campbell et al. (1998) recorded changes in track condition after only 20 foot transits. Tejedo et al. (2009), in a carefully controlled field trial on the Byers Peninsula, Livingston Island (also in the South Shetland Islands), noted that even a minimal human presence was sufficient to reduce collembolan abundance and that, on tracks, densities could be reduced to zero. Our data from Deception Island, where, unlike Tejedo et al.'s (2009) studies, Collembola were identified to species, showed that both NIS and native species are affected equally.

**Figure 3.**
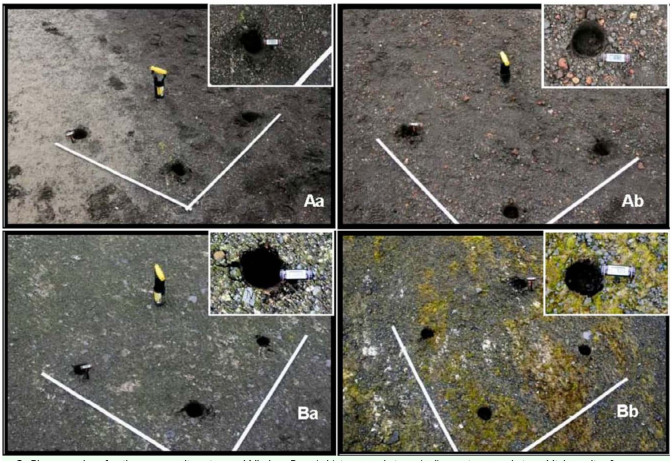
Photographs of soil core sampling sites at Whalers Bay. (a,b) impacted sites; (c,d) non-impacted sites. High quality figures are available online.

## Abbreviations

ASPA: Antarctic Specially Protected Area
BAS: British Antarctic Survey
NIS: non-indigenous species
